# Estimating the prevalence and incidence of treated type 2 diabetes using prescription data as a proxy: A stepwise approach on Iranian data

**DOI:** 10.1016/j.heliyon.2021.e07260

**Published:** 2021-06-09

**Authors:** Alireza Mirahmadizadeh, Sayed Aliakbar Banihashemi, Mehdi Hashemi, Sanaz Amiri, Suzan Basir, Alireza Heiran, Omid Keshavarzian

**Affiliations:** a Noncommunicable Diseases Research Center, Shiraz University of Medical Sciences, Shiraz, Iran; b Kazeroun Branch, College of Medicine, Islamic Azad University, Kazeroun, Iran; c Social Security Insurance, Shiraz, Iran; d Shiraz University of Medical Sciences, Shiraz, Iran; e Student Research Committee, School of Medicine, Shiraz University of Medical Sciences, Shiraz, Iran

**Keywords:** Prevalence, Diabetes mellitus, Prescription drug

## Abstract

**Aims:**

Type 2 diabetes is a serious health challenge, and large-scale studies on its prevalence in Iran are lacking. In pharmacoepidemiology, case-finding can be done by reviewing the prescription databases for specific drug(s) prescribed for a disease. We aimed to determine the prevalence and incidence of type 2 diabetes in Fars province, Iran, using prescription data and a stepwise approach to ascertain the results.

**Methods:**

A dataset of 3,113 insured individuals aged ≥35 years were selected. Their Prescription Data Centre records were reviewed for all drugs frequently used in controlling type 2 diabetes available in the Iranian pharmacopeia. Then we used a stepwise method for case-finding. In step one, each individual with a positive drug history for type 2 diabetes was labeled as an individual with diabetes. The next two steps were implemented for ascertainment of step one estimations.

**Results:**

Prevalence of type 2 diabetes based on prescription, internist opinion, and phone call verification in 2015 and 2016 was 9.3% and 10.3%, 8.5% and 9.8%, and 7.2% and 8.7%, respectively. An incidence of 1.9% was determined for 2016.

**Conclusions:**

We obtained a realistic estimation of prevalence and incidence of treated type 2 diabetes, using prescription data which are large-scale, low cost, and real-time.

## Highlights

• Prevalence of type 2 diabetes was 7.2% and 8.74% in 2015 and 2016.
• Prevalence of type 2 diabetes raised by 1.5% from 2015 to 2016.
• An incidence of 1.9% was determined for 2016.
• Both prevalence and incidence were higher in women.
• Metformin 500 and Glibenclamide 5 had equal or more than fair power (>70%) to identify the number of patients with type 2 diabetes.
• The stepwise method yields a realistic estimation on prevalence and incidence of treated type 2 diabetes, using prescription data which is large-scale, cheap and real-time.

## 1 Introduction

Type 2 diabetes is a major health challenge, affected approximately 424.9 million people worldwide in 2017, and this number is expected to rise to 628.6 million by 2045. This trend is more dramatic in the Middle East and North Africa (MENA) as it will be more than double by the same time [1, 2]. The Islamic Republic of Iran is one of the largest countries in MENA. In the latest International Diabetes Federation diabetes atlas, the prevalence of type 2 diabetes in Iranian adults was estimated to be 8.9% in 2017 [3].

Although several country level surveys in Iran were identified in literature, large-scale (nationwide or at providence level) representative data regarding the prevalence of type 2 diabetes and particularly its trend are scarce or even lacking. This information can contribute to the public health surveillance system to monitor real-time disease burden and to make decisions about healthcare resources and public health planning. In addition, current information is mostly obtained from relatively small studies in urban populations; hence, large-scale data covering both urban and rural areas will be advantageous [4, 5].

Prescription data can be the proxy for the epidemiological studies. The main assumption is that each person with a specific illness is expected to use a specific drug or list of drugs. Thus, case-finding can be potentially carried out by reviewing prescription databases, particularly disease specific drugs (drugs that are used for one or a limited number of disease). This type of study can be used to identify individuals with a diagnosis of type 2 diabetes, but estimation should be done with caution due to conditions with an overlapping drug list as well as several technical issues. These considerations will be discussed further.

**Subjects:** The present study is the first study of its type to use prescription data to calculate the prevalence and incidence of type 2 diabetes in Fars province, Iran, and it utilizes a stepwise approach to yield more accurate results.

## 2 Methods and materials

### 2.1 Sample selection

Similar to many countries, Iran does not have an electronic integrated prescription and health insurance platform. However, the most extensive data belong to the social security organization as it covers approximately half of the Iranian population [6, 7]. Since other health care providers lacked an applicable electronic prescription platform at the time of our study or in some access was denied, we did not include beneficiaries from other health care providers like military staff, bank employees, petroleum industry staff, etc. as well as their families or the uninsured population. In this cross-sectional study, from March 21, 2015 to March 20, 2017, a list of all insured individuals was acquired from the social security organization branch of Fars province. All of these individuals were aged ≥35 years and each one had a family physician who had been registered in the family physician office of Shiraz University of Medical Sciences, Shiraz, Iran.

That list identified 875,502 unique national ID (number of individuals), and we selected a dataset of 3,100 individuals. The sample size was calculated by assuming variables as 1-*α* = 95% (corresponding *Z* of 1.96), prevalence = 10%, precision of the estimate (d) = 0.013, and design effect (DEFF) = 1.5.

$$Sample\;size=\;\frac{{Z_{1-\alpha /2}}^{2}\times p\times \left(1-p\right)}{d^{2}}\;\times \;DEFF$$

An individual was included if they were insured by the social security organization, resided in Fars province, aged ≥35 years on March 21, 2015 (equivalent to the first day of solar Hijri calendar), and had prescription data in the data centre. Cases in each age and gender group were selected using a proportionate stratified random number selection function in Microsoft Excel data analysis. Proportions were obtained from the latest national population and housing census report of Statistical Centre of Iran published in 2016 [8].

In the test dataset (3,100 individuals), an individual was excluded if no data was available- almost all due to migration. To discriminate these individuals from those who had been deceased or were considered as healthy without prescription data during the study period, we also checked their data to find the exact reason. Also, those who were selected for the last step of data gathering were excluded if their phone number was not available or had been changed, or they were not cooperative. To maintain the target samples size excluded individuals were replaced using the same random selection and exclusion processes.

This study was approved by Shiraz University of Medical Sciences Local Ethics Committee (code: IR.SUMS.REC.1396.S229).

### 2.2 Data acquisition and case-finding method

The test dataset contained age, gender, phone number, insurance number, and national ID. Initially, de-duplication was carried out, by checking the abovementioned variables as well as prescription date and drug content. Records in the prescription data base were reviewed for all drugs frequently used in controlling type 2 diabetes listed in the Iranian pharmacopeia; these include metformin, glibenclamide, gliclazide, pioglitazone, acarbose, repaglinide, sitagliptin, and different types of insulin.

**Figure 1:**
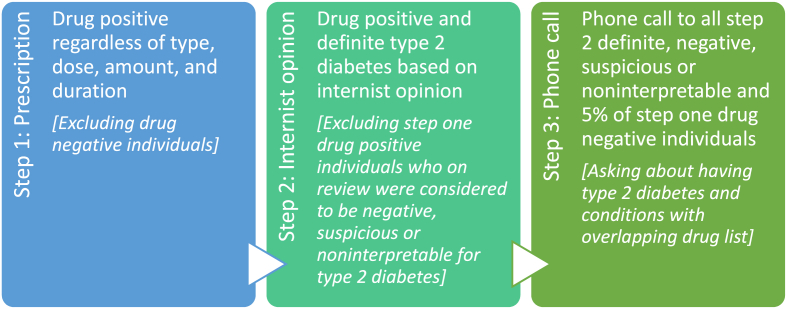
Stepwise case-finding method

We used a stepwise method for the case-finding since anti-diabetic drugs were not specific for type 2 diabetes and might have increased the false positive rate. Our approach was expected to estimate more accurate data as it progressed (Figure 1). In step one, each individual with a positive drug history for type 2 diabetes, regardless of type, dose, amount, and duration of prescription, was identified as an individual with diabetes. Steps two and three were implemented for ascertainment. In step two, prevalence was obtained, seeking the opinion of two independent internists in the field of diabetes by tracking the pattern and amount of drug administration and requested paraclinical tests. The sample population was divided into four groups: (1) drug negative – diabetes negative, (2) drug positive – diabetes negative, (3) drug positive – diabetes suspicious or non-interpretable, and (4) drug positive – diabetes definite. Group four represented the prevalence estimation. In step three, to maximize the accuracy, a phone call was made to 5% of all individuals in group one and all individuals in groups two, three, and four. They were asked about their drugs, types of diabetes (type 1 diabetes, type 2 diabetes, gestational diabetes, or prediabetes), presence of polycystic ovary syndrome (35–50 years old women), fatty liver and dieting. Agreement Kappa measurement and standard error (SE) on prevalence estimation of type 2 diabetes between the three steps is shown in Table 1.

**Table 1:** Agreement Kappa measurement (SE) on accuracy of prevalence estimation of type 2 diabetes between prescription, internist opinion, and phone call during 2015–2016.

	By Prescription		By Internist		By Telephone	
By Prescription		2016→	0.95 (0.01)		0.82 (0.02)	
	2015↓					
By Internist	0.95 (0.01)			2016↑	0.83 (0.02)	
			2015↓			
By Telephone	0.85 (0.02)		0.88 (0.02)			2016↑
					2015←	

### 2.3 Statistical analysis

Statistical analysis was performed using the statistical package for social sciences (SPSS) (IBM Corp. Released 2013. IBM SPSS Statistics for Windows, Version 22.0. Armonk, NY: IBM Corp.) and Microsoft Excel data analysis software. Quantitative and qualitative variables were described using mean ± standard deviation (SD) and frequency (%), respectively. Also, the Monte Carlo statistic was used to calculate the 95% confidence interval (CI) for prevalence and incidence estimates. Incidence was obtained by identifying those who had anti-diabetic drugs only in the second year of the study [9].

We studied the sensitivity, specificity, positive predictive value (PPV), and negative predictive value (NPV) of each anti-diabetes drug to identify cases of diagnosed diabetes by using the number of consumed tablets in one year (predicted value). To do so, the data from step 3 were considered as the reference (observed value). Then, sensitivity, specificity, PPV, and NPV at the optimal value of the number of consumed tablets in one year were reported for each anti-diabetes drug. In addition, the area under the curve (AUC) of the significant drugs according to a receiver operating characteristic (ROC) analysis was reported.

The Post-Hoc sensitivity analysis was done by Microsoft Excel data analysis to assess the effect of uncertainty on estimation. Variables that might introduce uncertainty were ascertained from the literature or phone conversations (retrieved from step three); they were prevalence of polycystic ovary syndrome, prevalence of fatty liver, dieting, gestational diabetes, type 1 diabetes, prediabetes, prescription for another person, individuals with diabetes who were contraindicated or did not use metformin, and inability to determine the type of diabetes by means of prescription. Worth noting, sensitivity analysis showed that the effect of uncertainties was negligible (~0.01%). *P*-value ≤ 0.05 was considered statistically significant.

## 3 Results

### 3.1 Basic information

The total number of individuals in the adult list group was 875502- of which 198827, 130800, 147960, 71441, 101558, 113290, and 111626 were in the age groups 35–39, 40–44, 45–49, 50–54, 55–59, 60–69, and ≥70 years, respectively. According to the corresponding proportion in each group, the sample size was calculated as 707, 465, 526, 254, 361, 403, and 397 for 35–39, 40–44, 45–49, 50–54, 55–59, 60–69, and ≥70 years age groups, respectively.

The mean age of 3,113 included individuals was 51.5 ± 13.6 year (range 35–101), and 50.1% were female. The mean yearly drug prescription (each drug in the prescription- each line was defined as 1 prescription) for each individual was 31 ± 42.7 (range 0–405, total: 96588) in 2015, and 32.6 ± 49.1 (range 0–580, total: 101570) in 2016, respectively. The frequency of anti-diabetes drug prescriptions among all records of the 3,113 individuals in the dataset was 4% (3829 out of 96588) in 2015 and 4.4% (4455 out of 101570) in 2015; moreover, the largest proportion belonged to Metformin 500 (2015 = 1.8% [1722 out of 96588], 2016 = 2% [1995 out of 101570]) and Glibenclamide 5 (2015 = 0.9% [834 out of 96588], and 2016 = 8% [846 out of 101570]).

Table 2 depicts characteristics of each anti-diabetes drug among its users during 2015–16. In both studied years, Metformin 500, Glibenclamide 5, Acarbose 50, Gliclazide 80, and Acarbose 100 were prescribed for more individuals than any other anti-diabetes drugs. Compared to 2015, most of the anti-diabetes drugs were consumed by more individuals in 2016.

**Table 2:** List of prescribed anti-diabetes drugs among insured individuals during 2015–16.

Drug	2015			2016			Changing rate (%)^4^
	No. (%)^1^	SUM^2^	M±SD^3^	No. (%)^1^	SUM^2^	M±SD^3^	
Metformin 500	236 (7.6%)	214410	908.5 ± 970	268 (8.6%)	258140	963.2 ± 1101	13.6%
Glibenclamide 5	118 (3.8%)	100820	854.4 ± 990	118 (3.8%)	101120	857 ± 1002	0%
Acarbose 50	44 (1.4%)	22578	513.1 ± 502	50 (1.6%)	34475	689.5 ± 846.3	13.6%
Acarbose 100	33 (1.1%)	16556	501.7 ± 461	29 (0.9%)	17750	612 ± 870.6	-12.1%
Gliclazide 80	27 (0.9%)	21300	788.9 ± 1058	40 (1.3%)	22380	559.5 ± 618	48.1%
Pioglitazone 30	24 (0.8%)	9000	375 ± 308.1	31 (1%)	9460	305.1 ± 263.7	29.2%
Pioglitazone 15	20 (0.6%)	5864	293.2 ± 221	24 (0.8%)	11050	460.4 ± 429.4	20%
Insulin (Glargine)	17 (0.5%)	852	50.1 ± 29.1	19 (0.6%)	1407	74 ± 73.3	11.8%
Insulin (Aspart)	12 (0.4%)	624	52 ± 52.4	16 (0.5%)	880	55 ± 54.6	33.3%
Insulin (NPH)	10 (0.3%)	120	12 ± 16.3	6 (0.2%)	68	11.3 ± 4.1	-40%
Metformin 1000	5 (0.2%)	2440	488 ± 465	5 (0.2%)	1240	248 ± 137	0%
Repaglinide 1	5 (0.2%)	950	190 ± 78.1	5 (0.2%)	2820	564 ± 555.4	0%
Insulin (Regular)	5 (0.2%)	56	11.2 ± 16.2	3 (0.1%)	21	7 ± 2.6	-40%
Repaglinide 2	3 (0.1%)	3400	1133 ± 923.7	4 (0.2%)	2750	687.5 ± 592.1	-33.3%
Repaglinide 0.5	2 (0.1%)	320	160 ± 56.5	1 (0.0%)	60	60 ± 0	-
Met. + Glib.‡	1 (0.0%)	720	720 ± 0	1 (0.0%)	900	900 ± 0	-
Pioglitazone 45	1 (0.0%)	200	200 ± 0	1 (0.0%)	400	400 ± 0	-
Insulin (Biphasic)	1 (0.0%)	4	4 ± 0	0 (0%)	0	0	-
Any insulin	30 (1%)	†	†	30 (1%)	†	†	0%
No drug	2822 (90.1%)	†	†	2791 (89.7%)	†	†	1.1%
1 drug	129 (4.01%)	†	†	146 (4.7%)	†	†	13.2%
2 drugs	93 (3%)	†	†	95 (3.1%)	†	†	2.1%
3 drugs	40 (1.3%)	†	†	51 (1.6%)	†	†	27.5%
4 drugs	19 (0.6%)	†	†	21 (0.7%)	†	†	10.5%
5 drugs	7 (0.2%)	†	†	6 (0.2%)	†	†	-14.3%
6 drugs	3 (0.1%)	†	†	2 (0.1%)	†	†	-33.3%
7 drugs	0 (0%)	†	†	1 (0.03%)	†	†	-

1 Number (%) of each drug users among 3,113 individuals.

2 Sum of drug tablets (or another modality) prescribed in 2015 or 2016.

3 Mean (±SD (standard deviation)) prescribed drug tablets (or another modality) of users in 2015 or 2016.

4 Changing rate (%) from 2015 to 2016 in number of each drug users.

† Not applicable.

‡ Metformin + Glibenclamide.

### 3.2 Prevalence estimation statistics

**Figure 2:**
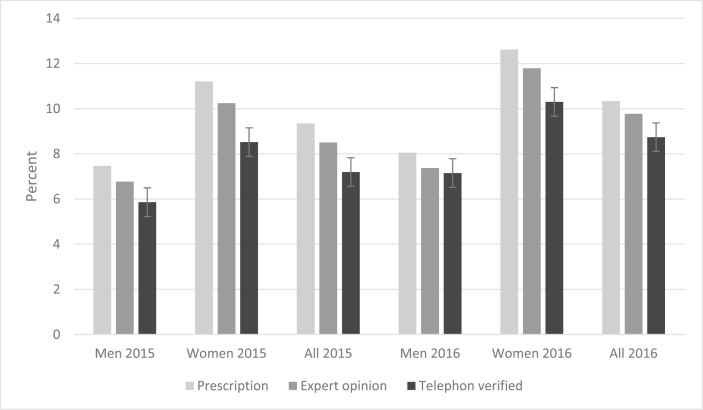
Prevalence of type 2 diabetes based on prescription, internist opinion and phone call verification in 2015 and 2016.

Prevalence of type 2 diabetes based on prescription, internist opinion, and phone call verification in 2015 and 2016 was 9.3% and 10.3%, 8.5% and 9.8%, and 7.2% and 8.7%, respectively (Table 3). The constant decrease from step 1 to step 3 implied false positives. Furthermore, as Post-Hoc sensitivity analysis result was not significant, we believe that the more accurate estimates could be attributed to the removal of false positives in steps two and three (Figure 2).

**Table 3:** Type 2 diabetes prevalence estimates in each step during 2015–16.

Prevalence of type 2 diabetes	2015		2016	
	No.	Prevalence (95% CI)	No.	Prevalence (95% CI)
By prescription				
Male	116	7.5 (6.3–8.9)	125	8.1 (6.8–9.5)
Female	175	11.2 (9.7–12.9)	197	12.6 (11.1–14.4)
All	291	9.3 (8.4–10.4)	322	10.3 (9.3–11.5)
By two independent internists				
Male	105	6.8 (5.6–8.1)	120	7.7 (6.5–9.2)
Female	160	10.2 (8.8–11.8)	184	11.8 (10.3–13.5)
All	265	8.5 (7.6–9.5)	304	9.8 (8.8–10.9)
By phone call				
Male	91	5.9 (4.8–7.1)	111	7.2 (6–8.5)
Female	133	8.5 (7.2–10)	161	10.3 (8.9–11.9)
All	224	7.2 (6.3–8.2)	272	8.7 (7.8–9.8)

The prevalence was higher in women than men in all age groups (Figure 2). In all three steps in both 2015 and 2016, the prevalence was higher in the older age groups. It should be noted that a relatively lower prevalence in the ≥70 years age group, compare to 60–69 and 55–59 age groups, might be explained by a higher mortality rate (Table 4).

**Table 4:** Type 2 diabetes prevalence estimates by age groups in each step during 2015–16.

Prevalence of type 2 diabetes	2015		2016	
	No.	Prevalence (95% CI)	No.	Prevalence (95% CI)
By prescription				
35–39 (n = 707)	28	4 (2.7–5.7)	21	3 (1.9–4.5)
40–44 (n = 465)	10	2.1 (1.2–3.9)	8	1.7 (0.9–3.4)
45–49 (n = 526)	35	6.6 (4.8–9.1)	42	8 (6–10.6)
50–54 (n = 254)	33	13 (9.4–17.7)	41	16.1 (12.1–21.2)
55–59 (n = 361)	57	15.8 (12.4–19.9)	59	16.3 (12.9–20.5)
60–69 (n = 403)	72	17.9 (14.4–21.9)	87	21.6 (17.9–25.9)
+70 (n = 397)	56	14.1 (11–17.9)	64	16.1 (12.8–20.1)
By internist opinion				
35–39 (n = 707)	18	2.5 (1.6–4)	18	2.5 (1.6–4)
40–44 (n = 465)	9	1.9 (1–3.6)	8	1.7 (0.9–3.4)
45–49 (n = 526)	34	6.5 (4.7–8.9)	37	7 (5.1–9.5)
50–54 (n = 254)	29	11.4 (8.1–15.9)	40	15.7 (11.8–20.7)
55–59 (n = 361)	52	14.4 (11.2–18.4)	57	15.8 (12.4–19.9)
60–69 (n = 403)	70	17.4 (14–21.4)	82	20.3 (16.7–24.5)
+70 (n = 397)	53	13.3 (10.3–17)	62	15.6 (12.4–19.5)
By phone call				
35–39 (n = 707)	9	1.3 (0.7–2.4)	10	1.4 (0.8–2.6)
40–44 (n = 465)	8	1.7 (0.9–3.4)	9	1.9 (1–3.6)
45–49 (n = 526)	23	4.4 (2.9–6.5)	25	4.7 (3.2–6.9)
50–54 (n = 254)	27	10.6 (7.4–15)	34	13.4 (9.7–18.1)
55–59 (n = 361)	49	13.6 (10.4–17.5)	59	16.3 (12.9–20.5)
60–69 (n = 403)	61	15.1 (12–19)	76	18.9 (15.3–23)
+70 (n = 397)	47	11.8 (9–15.4)	59	14.9 (11.7–18.7)

### 3.3 Incidence in 2016 and study on the trend of prevalence based on step three results

Prevalence of type 2 diabetes increased by 1.5% from 2015 to 2016 and its incidence was 1.9% in 2016 (Table 5). Incidence was higher in women compared to men and increased with increasing age group; the highest incidence was observed in the 60–69 age group (Table 6).

**Table 5:** Prevalence and incidence of type 2 diabetes based on step 3 during 2015–16.

	**Year 2016**	**Yes**	**No**	**Total**
**Year 2015**				
**Yes**		216	8^a^	**224**^b^**(7.2%)**
**No**		56^c^ (1.9%)	2833 (98.1%)	**2889 (92.8%)**
**Total**		**272**^d^**(8.7%)**	**2481 (91.3%)**	**3113 (100%)**

a Individuals considered to have type 2 diabetes in 2015 but had no prescription drug data for 2016, i.e. untreated or died. Unavailable or uncooperative individuals as well as those with changed phone number are not included.

b Prevalence of type 2 diabetes in 2015.

c Newly diagnosed type 2 diabetes and consider as incidence during in 2016.

d Prevalence of type 2 diabetes in 2016.

**Table 6:** 2016 incidence of type 2 diabetes by sex and age groups based on step 3 data.

Variable	Incidence (95%CI)
**Sex**	
- Male	1.5 (1–2.2)
- Female	2.1 (1.1–2.9)
**Age groups (year)**	
- 35-39	0.3 (0–1)
- 40-44	0.2 (0–1.2)
- 45-49	1.1 (0.5–2.5)
- 50-54	3.5 (1.9–6.6)
- 55-59	2.8 (1.5–5)
- 60-69	3.7 (2.3–6)
- +70	3.3 (1.9–5.5)

We studied the sensitivity, specificity, positive predictive value (PPV), and negative predictive value (NPV) of each anti-diabetes drug in identifying type 2 diabetes by using the number of consumed tablets in one year. To do so, the results of step 3 were considered as the reference. Then, sensitivity, specificity, PPV, and NPV at the optimal value of the number of consumed tablets in one year were reported for each anti-diabetes drug. In addition, the area under the curve (AUC) of the significant drugs according to receiver operating characteristic (ROC) analysis was reported.

### 3.4 Diagnostic test performance of drugs in identifying type 2 diabetes based on step three results

**Figure 3:**
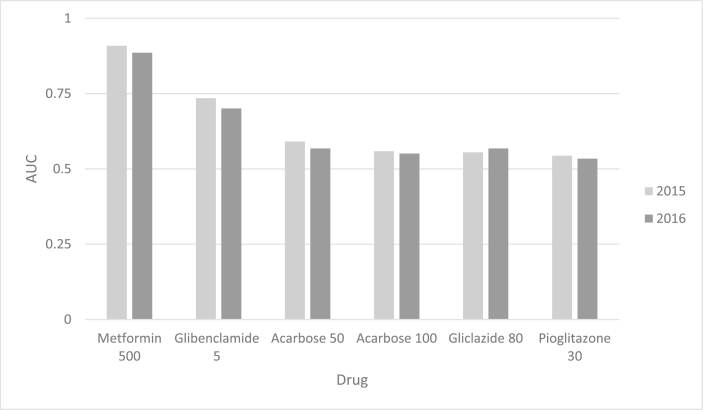
Area under the curve (AUC) of significant drugs according to ROC analysis.

Considering both 2015 and 2016 results, the highest positive predictive value was related to Insulin Glargine, Acarbose 50, and Gliclazide 80. The highest negative predictive value was related to Metformin 500, Glibenclamide 5, and Acarbose 50 (Table 7). Also, Table 8 and Figure 3 show the predictive value of consumed tablets of each drug in one year in predicting type 2 diabetes. Among all drugs, Metformin 500 and Glibenclamide 5 had equal or more than fair diagnostic power (>70%) to identify type 2 diabetes in our test dataset.

**Table 7:** Positive predictive value (PPV) and negative predictive value (NPV) of each drug in identifying type 2 diabetes using step three as the reference, during 2015–16.

Drug	2015		2016	
	PPV (95% CI)	NPV (95% CI)	PPV (95% CI)	NPV (95% CI)
Acarbose 100	81.8 (65.6–91.4)	93.6 (92.7–94.4)	96.5 (82.8–99.4)	92 (91.1–93)
Acarbose 50	93.2 (81.8–97.6)	94 (93.1–94.8)	94 (83.8–97.9)	92.6 (91.7–93.5)
Glibenclamide 5	89.8 (83.1–94.1)	96 (95.3–96.8)	93.2 (87.2–96.5)	94.6 (93.7–95.4)
Gliclazide 80	92.6 (76.6–97.9)	93 (92.6–94.4)	92.5 (80.1–97.4)	92.3 (91.4–93.2)
Insulin (Aspart)	100 (75.7–100)	93.2 (92.2–94.1)	87.5 (64–96.5)	91.7 (90.6–92.6)
Insulin (Biphasic)	100 (20.6–100)	92.8 (91.8–93.7)	-	-
Insulin (Glargine)	94.1 (73.1–98.9)	93.3 (92.4–94.1)	94.7 (75.4–99.1)	91.8 (90.8–92.7)
Insulin (NPH)	70 (39.7–89.2)	93 (92.1–93.8)	100 (60.9–100)	91.4 (90.4–92.4)
Insulin (Regular)	60 (23.1–88.2)	92.1 (91.9–93.7)	100 (43.9–100)	91.3 (90.3–92.3)
Any insulin	86.7 (70.3–94.7)	93.8 (92.7–94.4)	93.3 (76.7–98.2)	92.1 (91.1–92.9)
Metformin 1000	80 (37.6–96.4)	92.9 (92–93.8)	100 (56.6–100)	91.4 (90.4–92.3)
Metformin 500	78.4 (72.7–83.2)	98.6 (98.2–99)	74.1 (68.8–78.9)	97.9 (97.3–98.4)
Met. + Glib.‡	100 (20.7–100)	92.8 (91.9–93.7)	100 (20.7–100)	91.3 (90.3–92.2)
Pioglitazone 15	85 (63.9–94.8)	93.3 (92.4–94.1)	79.2 (59.5–90.8)	91.8 (90.8–92.7)
Pioglitazone 30	83.3 (64.2–93.3)	93.4 (92.9–94.2)	83.9 (67.4–92.9)	92 (91.1–92.9)
Pioglitazone 45	100 (20.7–100)	92.8 (91.9–93.7)	100 (20.7–100)	91.3 (90.3–92.2)
Repaglinide 0.5	100 (34.2–100)	92.9 (91.9–93.7)	100 (20.7–100)	91.3 (90.3–92.2)
Repaglinide 1	100 (56.6–100)	92.9 (92–93.8)	100 (56.6–100)	91.4 (90.37–92.3)
Repaglinide 2	100 (43.9–100)	87.3 (86.1–88.4)	75 (30.1–95.4)	91.3 (90.3–92.3)
No drug	†	98 (97.5–98.5)	†	99.2 (98.8–99.5)
1 drug	56.6 (48–64.8)	†	57.5 (49.4–65.3)	†
2 drugs	84.9 (76.3–90.8)	†	89.5 (81.7–94.2)	†
3 drugs	95 (83.5–98.6)	†	98 (89.7–99.7)	†
4 drugs	94.7 (75.4–99.1)	†	100 (84.5–100)	†
5 drugs	85.7 (48.7–97.4)	†	100 (61–100)	†
6 drugs	100 (43.8–100)	†	100 (34.2–100)	†
7 drugs	-	†	100 (20.6–100)	†

† Not applicable.

‡ Metformin + Glibenclamide.

**Table 8:** Area under curve (AUC) of significant anti-diabetes drugs according to ROC analysis.

Drug	year	AUC	SE	95% CI	*p*-value	Cut-off-point (tablet #)
Metformin 500	2015	0.91	0.01	0.88–0.94	<0.0001	90
	2016	0.89	0.01	0.86–0.92	<0.0001	25
Glibenclamide 5	2015	0.73	0.02	0.69–0.78	<0.0001	50
	2016	0.7	0.02	0.66–0.74	<0.0001	35
Acarbose 50	2015	0.59	0.02	0.55–0.63	<0.0001	15
	2016	0.57	0.02	0.55–0.62	<0.0001	15
Acarbose 100	2015	0.56	0.02	0.52–0.60	0.005	50
	2016	0.55	0.02	0.51–0.59	0.006	30
Gliclazide 80	2015	0.55	0.02	0.51–0.6	0.001	50
	2016	0.57	0.02	0.53–0.61	<0.0001	15
Pioglitazone 30	2015	0.54	0.02	0.5–0.59	0.032	30
	2016	0.53	0.02	0.5–0.57	0.06	30

## 4 Discussion

Prescription data can be the proxy for the epidemiological studies [10, 11]. A great deal of such studies have been conducted in Europe, especially Nordic countries with more than twenty articles from 2004 to 2009, using the Nordic prescription databases [12]. First-in-class studies date back to the late 1980s [13]; and now, a considerable number of studies can be found in the literature regarding several conditions like type 1 and type 2 diabetes [4, 13, 14, 15], multiple sclerosis [16, 17, 18, 19, 20], Parkinson's disease [21, 22, 23, 24, 25], epilepsy [26, 27], chronic kidney disease [28], asthma [29, 30], atopic dermatitis and allergic rhino conjunctivitis [31], heart disease [32], and so forth. To the best of our knowledge, the present study is the first study of its type using prescription data to calculate the prevalence and incidence of type 2 diabetes in Fars province, Iran. We also applied our novel model to yield a more accurate case-finding approach.

### 4.1 Main findings of the present study

The main finding of our study is the prevalence of treated type 2 diabetes equal to 7.2% and 8.7% amongst Fars province adults in 2015 and 2016, respectively. An incidence of 1.9% was determined for 2016. There are only two recent publications on the prevalence of type 2 diabetes in Fars province and both were conducted among rural populations with noticeable methodological and study setting differences when compared with the present study; hence, we cannot compare their results to our estimates. The first study showed a prevalence of 15.8% diagnosed type 2 diabetes amongst 1523 individuals aged ≥30 years during 2015–2016 [33]. The second study was conducted in a large sample of 447,251 individuals aged ≥30 years during 2008–2009. The prevalence of diagnosed type 2 diabetes was 12.3% [33]. In line with our study, Faramarzi et al. [34] reported the female dominant pattern.

### 4.2 What is already known about this topic

An epidemiological study through prescription data has some limitations: (1) Such data can be used only for disease specific drugs. Moreover, several adjustments should be made to decrease the overestimation; (2) This method is not sensitive to identifying diabetic patients who only control their diabetes with lifestyle modifications, do not use anti-diabetic drugs, or are undiagnosed [35,36]; (3) Human errors such as forgetfulness and pressure of work in pharmacies can cause under-registration [35]; (4) Self-funding or over-the-counter use can cause underestimation, especially in diseases accompanied by low-cost drugs. On the other hand, the poor drug coverage in a costly disease such as some malignancies (Cyclophosphamide is an example in Iran) can cause the same problem [37, 38].

Despite the above-mentioned limitations, prescription data have numerous advantages: (1) This method is less likely to under-report disease prevalence and is accurate since it is used for administrative purposes [4,39]; (2) It allows the study of disease prevalence on a large-scale, unaffected by between-area variations (both urban and rural populations) [5]; (3) Prescription data is a spinoff of health system, which is not costly and is readily available, so it can be a preferred method in low resource areas; (4) They yield the real-time data to track the prevalence or incidence; (5) They lack selection bias, particularly when the database captures the annual prescription records for each patient [40]. Since our data was limited to a single source and might have excluded certain socioeconomic groups (those with other health insurances and those without health insurance), it might exert selection bias in our study, particularly; (6) They are sensitive to change in diagnostic criteria or prescription pattern; (7) Owing to the high coverage, patients with diseases that are treated with the so-called “orphan drugs” can be identified [41]; (8) Compared to methods like self-declaration of a disease, prescription data are less prone to over-reporting in conditions that are diagnose clinically (i.e. rheumatologic diseases) or have a vague definition i.e. migraine, depression, or anxiety [38, 42, 43, 44].

### 4.3 What our method adds

In line with the previous studies, individuals prescribed any type of anti-diabetic drugs were labelled to have diabetes. Our proposed stepwise method tried to obtain a more realistic estimation. Reporting the usefulness of each anti-diabetic drugs in identifying the number of patients with type 2 diabetes was a novelty of our work, which was made possible by step three [41]. Noticeably, it appears that the results are reliable. For example, metformin had an excellent and the highest NPV since almost all the patients with type 2 diabetes use metformin, as well as, a fair PPV because it is used in other medical conditions.

Also, we showed how to estimate the incidence of type 2 diabetes. Worth noting, our results must be carefully interpreted. For example, prescription data only represents drug-treated patients and therefore slightly underestimated treated patients; since we showed that NPV of “no drug” was not 100% (98% and 99.2% in 2015 and 2016, respectively). This negligible difference might be due to adding known type 2 diabetes patients in step three who were only on lifestyle modification. Also, it should be noted that estimating missed diabetic patients is challenging because it involves treated, untreated, and undiagnosed patients. Interestingly, the rate of missed diabetes can be estimated by combining prescription data with diagnosis information i.e. screening, diabetes registries, door-to-door or self-declaration surveys, or reports from general practitioners, family physicians, or other health-care professionals [45].

It is noteworthy that prescription data have other potential uses for public health surveillance and health policy making. Currently, we are working on cost estimation of treatment regimens and laboratory data as a marker of follow up and treatment compliance.

### 4.4 Conclusion

In the present study, we tried to yield a realistic estimation of the prevalence and incidence of treated type 2 diabetes, using prescription data which are large-scale, low cost, and real-time. The prevalence of treated type 2 diabetes was 7.2% and 8.7% in 2015 and 2016. Comparing the statistics of the two consecutive years of the study, an increasing trend on the prevalence of type 2 diabetes was observed. We recommend that further studies are undertaken for other candidate diseases with nation-wide coverage and a longer study period to track epidemiological changes.

## Declarations

### Author contribution statement

Alireza Mirahmadizadeh: Conceived and designed the experiments; Analyzed and interpreted the data; Contributed reagents, materials, analysis tools or data.

Sayed Aliakbar Banihashemi: Conceived and designed the experiments; Analyzed and interpreted the data.

Mehdi Hashemi, Sanaz Amiri, Suzan Basir: Performed the experiments; Contributed reagents, materials, analysis tools or data.

Alireza Heiran: Conceived and designed the experiments; Analyzed and interpreted the data; Wrote the paper.

Omid Keshavarzian: Performed the experiments.

### Funding statement

This work was supported by the 10.13039/501100004320Shiraz University of Medical Sciences [grant No: 95-01-109-13675].

### Data availability statement

The authors do not have permission to share data.

### Declaration of interests statement

The authors declare no conflict of interest.

### Additional information

No additional information is available for this paper.
